# Optimality principles reveal a complex interplay of intermediate toxicity and kinetic efficiency in the regulation of prokaryotic metabolism

**DOI:** 10.1371/journal.pcbi.1005371

**Published:** 2017-02-17

**Authors:** Jan Ewald, Martin Bartl, Thomas Dandekar, Christoph Kaleta

**Affiliations:** 1 Research Group Theoretical Systems Biology, Department of Bioinformatics, Friedrich-Schiller-Universität Jena, Jena, Germany; 2 Department of Bioinformatics, Biocenter, Julius-Maximilians-Universität Würzburg, Würzburg, Germany; 3 Research Group Medical Systems Biology, Christian-Albrechts-Universität zu Kiel, Kiel, Germany; University of Toronto, CANADA

## Abstract

A precise and rapid adjustment of fluxes through metabolic pathways is crucial for organisms to prevail in changing environmental conditions. Based on this reasoning, many guiding principles that govern the evolution of metabolic networks and their regulation have been uncovered. To this end, methods from dynamic optimization are ideally suited since they allow to uncover optimality principles behind the regulation of metabolic networks. We used dynamic optimization to investigate the influence of toxic intermediates in connection with the efficiency of enzymes on the regulation of a linear metabolic pathway. Our results predict that transcriptional regulation favors the control of highly efficient enzymes with less toxic upstream intermediates to reduce accumulation of toxic downstream intermediates. We show that the derived optimality principles hold by the analysis of the interplay between intermediate toxicity and pathway regulation in the metabolic pathways of over 5000 sequenced prokaryotes. Moreover, using the lipopolysaccharide biosynthesis in *Escherichia coli* as an example, we show how knowledge about the relation of regulation, kinetic efficiency and intermediate toxicity can be used to identify drug targets, which control endogenous toxic metabolites and prevent microbial growth. Beyond prokaryotes, we discuss the potential of our findings for the development of antifungal drugs.

## Author summary

Understanding the guiding principles behind the evolution of metabolic networks and their regulation is of fundamental importance in a broad range of disciplines reaching from the identification of novel targets to treat infections to the utilization of microbial organisms in biotechnological production processes. In our study, we used an approach that allowed us to identify optimal regulatory strategies for the control of metabolic pathways in a scenario in which the intermediates of a metabolic pathway differ in their toxicity for the host organism. The results of our approach, whose validity we demonstrate through the large-scale analysis of pathway regulation in several thousand prokaryotic metabolic networks, show that toxic intermediates are often associated to strongly regulated enzymes. Additionally, transcriptional regulation preferably targets highly efficient enzymes thereby minimizing the effort in terms of protein production that is required to adjust the flux through a metabolic pathway. Moreover, in an example case, we show how knowledge about key targets of regulation can be used to identify novel antimicrobials that inhibit cellular growth through self-poisoning of the pathogen.

## Introduction

The consideration of organisms from the point of view of evolutionary adaptation is at the core of a large number of biological considerations [1–5]. The utilization of optimality principles that reflect forces of adaptation is a frequently used tool in Systems Biology to identify states in models of biological systems that are more likely and thereby drastically reduces the feasible solution space. Prominent examples of such methods are constraint-based modeling approaches which frequently use the notion of an optimal distribution of (metabolic) resources to maximize growth rate [6] to identify the biologically most plausible fluxes within a metabolic network.

While most optimization approaches employed in Systems Biology are considering optimality in a balanced (growth) state, the optimality in the dynamics of adaptation has recently received increasing attention [7–10]. While optimal growth in a particular condition is of high evolutionary advantage in constant environments, evolutionary theory posits that especially in changing environmental conditions those organisms that minimize the variance in fitness during environmental changes prevail [11, 12]. Minimization of variance in fitness in turn can be achieved through regulatory programs that allow organisms to quickly respond to environmental challenges [10, 13].

In previous works, we have identified a plethora of mechanisms and regulatory strategies by which organisms can reduce response times to adapt metabolic fluxes [10, 14, 15] or minimize the time required to produce protein complexes [16]. Concerning regulatory networks controlling metabolic pathways, we have previously established that with increasing protein costs, the complexity of such programs increases [10, 14]. Thus, pathways that require only small protein investment are frequently only controlled at a few key positions while all enzymes of a pathways are only regulated in a coordinated fashion, if pathway costs are high. The former mode of transcriptional control is referred to as sparse transcriptional regulation while the latter is called pervasive transcriptional regulation. For pathways with very high protein costs this even leads to the optimality of precisely timed activation programs targeting individual enzymes in a pathway [14].

An important problem that we did not consider in our previous works are differences in the toxicity of intermediates of metabolic pathways. Especially, after a change in environmental conditions, the adjustment of fluxes in a pathway can lead to a temporary build-up of intermediates [17, 18]. While we considered a generic upper bound on all metabolites previously, there are large differences in the side-effects that intermediates can exert. Especially for highly toxic intermediates, an accumulation needs to be avoided and a rapid conversion into downstream, probably less toxic intermediates, is required. For example the toxic intermediate homoserine, which is a precursor of the amino acids threonine, methionine and isoleucine, is tightly controlled by a complex interplay of transcriptional regulation and feedback inhibition mechanisms [18, 19]. In the other direction, the identification of specific regulatory programs that are utilized to avoid the accumulation of toxic intermediates is a promising avenue to discover novel endogenous antibiotics that allow to more efficiently kill pathogens through self-poisoning.

In this work, we use dynamic optimization to study how the toxicity of intermediates influences regulatory programs that control metabolic pathways. For such pathways, our optimization approach predicts a more focused regulation of enzymes that occur upstream of toxic intermediates. Moreover, there is a strong tendency of transcriptional regulation to target enzymes that are efficient in terms of catalytic activity as well as substrate affinity and possess less toxic upstream intermediates. Especially a focus on transcriptional regulation of efficient enzymes is surprising since it is often assumed that rate-limiting steps, which are catalyzed by inefficient enzymes, are key points of pathway control [20, 21]. We validate our prediction through a detailed examination of the interplay between enzyme efficiency as well as transcriptional control in *Escherichia coli* and a study of the relationship between regulatory effort as well as intermediate toxicity in the metabolic networks of more than 5000 prokaryotes. Moreover, we illustrate the usefulness of our approach through a detailed investigation of the influence of intermediate toxicity on the production of endogenous toxic metabolites in lipopolysaccharide metabolism.

## Materials and methods

### Modeling approach

**Fig 1:**
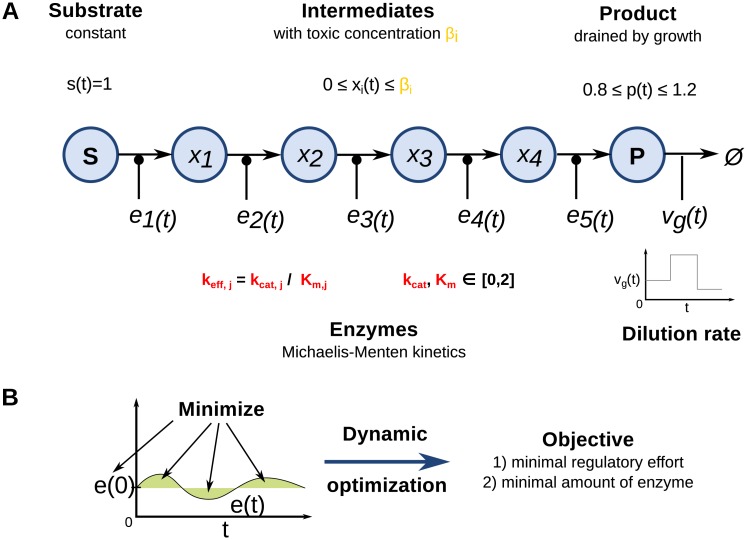
(A) Model of a linear pathway used for optimization. The substrate **S** is converted to a product **P** via five reactions catalyzed by enzymes with the concentration *e*_*j*_(*t*) with kinetic parameters *k*_*cat*_ and *K*_*m*_ and four intermediates *X*_*i*_ with concentration thresholds *β* that indicate their toxicity. The product **P** is diluted by *v*_*g*_ (dilution rate). (B) Objective function of the optimization problem. The objective function minimizes the regulatory effort of *e*(*t*) (green areas) and the initial concentration of enzymes *e*(0).

To model the toxicity of intermediates, we extended the optimization problem formulated by Wessely et al. [10] with the explicit consideration of toxicity of individual intermediates through concentration thresholds for each metabolite *β*_*i*_ (Fig 1A). The underlying ordinary differential equation system (ODE) describes a linear pathway converting a substrate *S* via five enzymatic reactions into a product *P* (see S1 Text). The selection of a five step linear pathway was made since it provides a good trade off between long and short linear pathways and is also the mean length of the defined linear pathways based on the listed pathways in MetaCyc [24]. Nevertheless, we obtain comparable results also for shorter and longer pathway lengths (see S2 Text).

While the substrate of the pathway is assumed to be constant in concentration, the product undergoes a drain e.g. by growth or other environmental changes *v*_*g*_(*t*), which vary over time:

$$\begin{array}{cc} v_{g}\left(t\right) & =\left\{\begin{array}{cc} g_{1}, & t<10\\ g_{2}, & 10\leq t<20\\ g_{3}, & 20\leq t\leq 30 \end{array}\right. \end{array}$$ (1)

The dilution is balanced by the regulation of the concentration of enzymes that catalyze the conversion of metabolites modeled as irreversible Michaelis-Menten kinetics with the parameters *k*_*cat*_ and *K*_*m*_. As discussed previously [10], considering an outflow of the product through growth is conceptually identical to a change of product demand, but can be formulated more conveniently in the optimization approach. Results were found to be robust against variations in the preset changes of dilution rate *v*_*g*_(*t*) (see S2 Text). Therefore the same changes in dilution rate *v*_*g*_(*t*) like in Wessely et al. [10] were used for all optimization runs.

The toxicity of intermediates is simulated as constraints on the concentration of the metabolites:

$$0\leq x_{i}\left(t\right)\leq \beta_{i}$$ (2)

A biological interpretation of the toxicity threshold used in the optimization is, for instance, the half inhibitory concentration (IC50) of a metabolite, that is, the concentration at which half of a bacterial population is inhibited in its growth.

In the objective function we minimize two factors: the regulatory effort and protein costs. The regulatory effort is modeled as the deviation from initial enzyme concentrations and protein costs by the initial concentration of enzymes. Both objectives are weighted with the factor *σ*. Hence, the following objective function:

$$\begin{array}{cc} F(e) & =\min_{e_{1\left(t\right),...,e_{5}\left(t\right)}}\;\sum_{j=1}^{5}\left(\int_{0}^{T_{max}}\left(\sigma \cdot \underset{\text{abundance}}{\underset{︸}{e_{j}\left(0\right)}}+\underset{\text{regulation}}{\underset{︸}{{\left(e_{j}\left(t\right)-e_{j}\left(0\right)\right)}^{2}}}\right)dt\right) \end{array}$$ (3)

is used to determine the optimal regulatory program. The factor *σ* models the costs for the whole pathway and not of single enzymes. In previous studies [14, 16] we discovered that varying synthesis costs within the pathway can influence the regulatory strategy, but is neglected here to focus on the impact of toxic intermediates.

The model consists of several parameters with arbitrary units (Table 1). The time-dependent concentrations of the five enzymes are used as control variables during the optimization. The simulation time span is set to 30 arbitrary time units and has no influence on optimization results (see also [15]). Parameters connected to toxicity (*β*) and kinetics (*k*_*cat*_,*K*_*m*_) are tested for their influence on optimal regulatory programs for two values of *σ* corresponding to high and low enzyme costs, respectively. To test the influence of toxicity and kinetic parameters, we performed 1000 runs in which their values were chosen randomly from the intervals indicated in Table 1.

**Table 1:** Parameter and variable overview.

Factor	Value	Description
*e*_*j*_	[0,∞]	enzyme concentration (control variable)
*σ*	$\frac{1}{3}$ or $\frac{1}{30}$	weight of enzyme cost
*T*_*max*_	30	timespan of optimization
*k*_*cat*,*j*_	[0,2]*	turnover number
*K*_*m*,*j*_	[0,2]*	inverse substrate affinity
*β*_*i*_	[0,4]*	threshold of intermediate concentrations

A star * indicates that the parameters are sampled from the indicated range.

### Dynamic optimization and analysis of simulation results

The above described optimization problem has time dependent and continuous decision (*e*(*t*)) and state variables (metabolite concentrations), and hence has to be solved using dynamic optimization. Similar to previous works [10, 14, 16], we used a quasi-sequential approach with the extensions of [22] to handle approximation errors and moving finite elements. Since this method is gradient-based, the optimization was repeated one hundred times for each random parameter set using random initializations to avoid local optima. For each random parameter set, we only considered the solution with the best objective function value.

To investigate the influence of kinetic and toxicity parameters on optimal regulatory programs, 1000 randomly distributed parameter samples were obtained as Sobol-sequences [23], which are values quasi-randomly distributed in the parameter space. In contrast to randomly chosen parameters sets, values obtained from a Sobol-sequence are maximizing the distance to each other in the parameter space. This leads to an optimal coverage of the parameter space and therefore less parameter tests are needed.

We are specifically interested in the relationship between regulatory effort, measured as deviation of enzyme time-courses from initial enzyme concentrations, and toxicity as well as kinetic parameters. To investigate this relationship, we partitioned the randomized parameters sets depending on the amount of regulatory effort at the individual pathway positions. Subsequently, we compared the distribution of toxicity parameters *β* and enzyme efficiency $k_{\text{eff}}=\frac{k_{cat}}{K_{m}}$ for cases with the 10% highest and lowest regulatory effort at individual pathway positions. To make regulatory effort comparable across the parameter samples, for each enzyme the fraction of regulation is normalized by dividing the regulatory effort with the sum of regulatory effort of all enzymes. Thus, the total normalized regulatory effort sums to one.

### Validation of optimization results in prokaryotes

**Fig 2:**
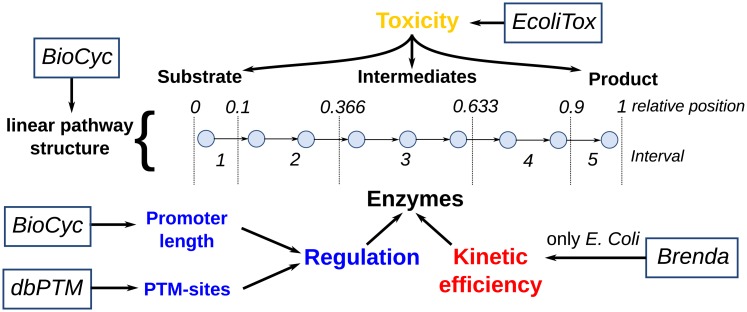
Overview of data and its processing for validation of optimization results. Rectangles depict the used databases and colors indicate, similar to the other figures, the considered pathway characteristics. A scheme of a 8-step linear pathway visualizes the binning into 5 intervals for the validation process.

As depicted in Fig 2, we validated regulatory programs predicted by the optimization approach using genomic data of pathways and the corresponding enzymes as well as compound toxicity from more than 5000 prokaryotic organisms represented in the BioCyc database (version 19.0) [24].

Pathway structures containing reactions and compounds were extracted from the MetaCyc database [24] and linear pathways were obtained as described previously [10, 15]. Since kinetic parameters are only available on a large-scale for a few model organisms, we validated the corresponding hypotheses only for *Escherichia coli* based on experimentally measured kinetic parameters from the Brenda database [25]. Only kinetic parameters without the term ‘mutant’ in the description of the experimental setup were considered.

As an estimate of regulatory effort targeted at individual enzymes, we used the promoter length and the number of predicted post-translational modification (PTM) sites as reference. We used predicted PTM-sites as estimator for post-translational regulation since they offer a view on non-transcriptional regulation. Predicted as well as experimentally validated PTM-Sites were obtained from dbPTM [26]. In a previous work we showed that promoter length, defined as intergenic distance between adjacent genes, is a good estimator of the number of transcription factors targeting a gene and hence of the complexity of the transcriptional regulatory network controlling this gene [15]. We showed this through a strong correlation between the number of transcription factors targeting a gene with its promoter length and a significant correlation between codon adaptation indices, a genomic measure of protein abundance [27], as well as promoter length (more abundant proteins tend to be controlled by more complex regulatory networks).

Since experimentally determined toxicity of compounds is only available for a small number of metabolites and organisms, the prediction tool EcoliTox was used [28]. This tool is based on a quantitative structure activity relationship (QSAR) model built on measured IC50 values, which is the concentration of a compound where 50% of the bacterial growth is inhibited. The QSAR-model was obtained through training of an classifier on a library of 166 molecules whose toxicity was experimentally determined in *E. coli*. Predicted logarithmized *IC*_50_ values for all compounds listed in the MetaCyc database were kindly provided by Dr. Pablo Carbonell.

We focused our analysis on sparsely regulated metabolic pathways, since, in contrast to pervasively regulated metabolic pathways, only the most important enzymes are under transcriptional control in such pathways and influence of intermediate toxicity is stronger. In accordance to our previous work [15], sparsely regulated pathways were defined at those with an average promoter length below 60% of the promoter length of non-metabolic genes of the same organism. Enzyme positions were normalized to a range of [0,1] by

$$relative\;position=\frac{position-1}{pathway\;length-1}$$ (4)

to make pathways comparable for different lengths. In order to apply the same approach of analysis as for the optimization results, enzymes were categorized into five intervals of their relative position, representing the five enzymes in the model (see Fig 2) and the average length of the used linear pathways (5.079). The intervals [0, 0.1] and [0.9, 1] contain mainly the first and the last enzyme and the three intervals in between were divided equally and correspond to the three intermediate enzymes. The binning of pathways into intervals was also made for different pathway lengths (three and seven steps), but was found either to short to resolve the linear pathway characteristics (for short pathways) or not to differ in results if more intervals were used (see S2 Text).

Promoter lengths, number of PTM-sites and *log* IC50-values were Z-transformed to make data comparable across different pathways and organisms:

$$Z\left(x\right)=\frac{x-avg}{sd}$$ (5)

where *Z*(*x*) is the transformed, *avg* the mean and *sd* the standard deviation of the corresponding values *x* across the entire pathway.

To match the analysis of the optimization results, we defined strongly or weakly regulated intervals of linear pathways as those where the mean regulatory effort of the interval was among the 10% highest (strong regulation) or lowest (weak regulation) across all pathways. Results show similar tendencies and significance for higher thresholds (25%).

Subsequently, the underlying pathway characteristics, like intermediate toxicity, of strongly and weakly regulated pathways were compared for each interval (see S2 Fig) or intervals are condensed regarding the position of strongly or weakly regulated enzymes to directly validate the hypotheses. For the latter, intervals are combined before and after the strongly regulated enzyme, if positions of strongly regulated enzymes are not the reference regulatory strategy involving the first and the last enzyme.

## Results

### Optimization of a linear metabolic pathway model considering toxic intermediates

**Fig 3:**
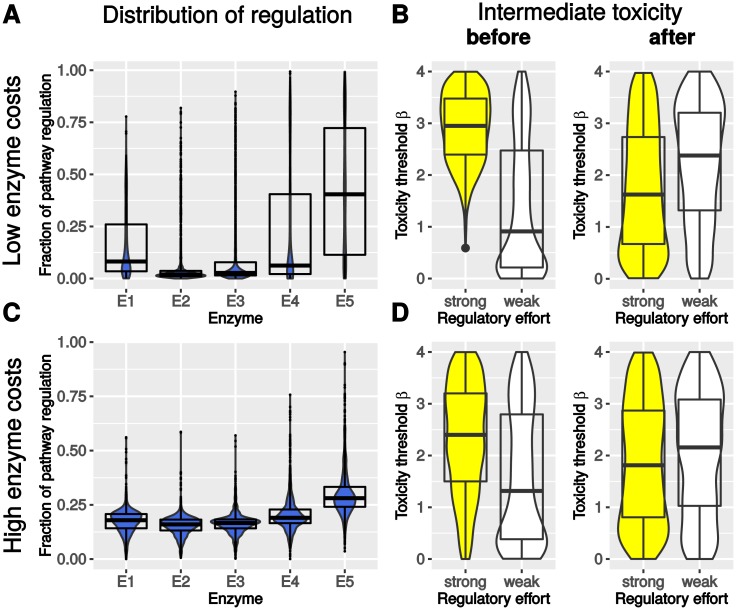
Relationship between regulatory effort and intermediate toxicity for low (A,B) and high enzyme costs (C,D). (A,C) The fraction of regulation is displayed in blue for each enzyme. (B,D) The intermediate toxicity is depicted in yellow (white) for the case of a strongly (weakly) regulated enzyme and the toxicity threshold of intermediates before and after (lower values indicate higher toxicity). All plots show the distribution of values as a combination of violin- and box-plots indicating median and 25–75% percentile.

In a first step, we analyzed the influence of the inclusion of toxicity constraints on general properties of pathway regulation. To this end, we determined the relative distribution of regulatory effort for each pathway position across the 1000 randomly sampled parameter combinations for the model including toxicity constraints. In accordance to our previous results [10], the regulatory effort was mainly targeted at the initial and the terminal enzyme for low protein costs ($\sigma =\frac{1}{30}$, Fig 3A), corresponding to a sparse transcriptional regulation. For high enzyme costs ($\sigma =\frac{1}{3}$, Fig 3C) we observe a rather uniform distribution of regulatory effort across the entire pathway, corresponding to a pervasive transcriptional regulation. Interestingly, the variation is stronger under the scenario of low enzyme costs (± 26.0%) suggesting that the influence of parameters on the regulatory strategy is greater compared to high enzyme costs (± 8.9%) and the position of strongly regulated enzymes changes depending on the pathway characteristics.

To better understand the influence of parameters on the targets of regulation, we separated all parameter samples into cases with weak and strong regulation for the individual pathway positions (upper and lower 10% percentile) and compared the distribution of the corresponding parameter samples between those cases (see Material and Methods). Based on this separation, we compared toxicity (Fig 3B and 3D) and kinetic parameters across all enzymes for cases with strong or weak regulation at individual pathway positions (see S1 Fig for detailed plot).

**Fig 4:**
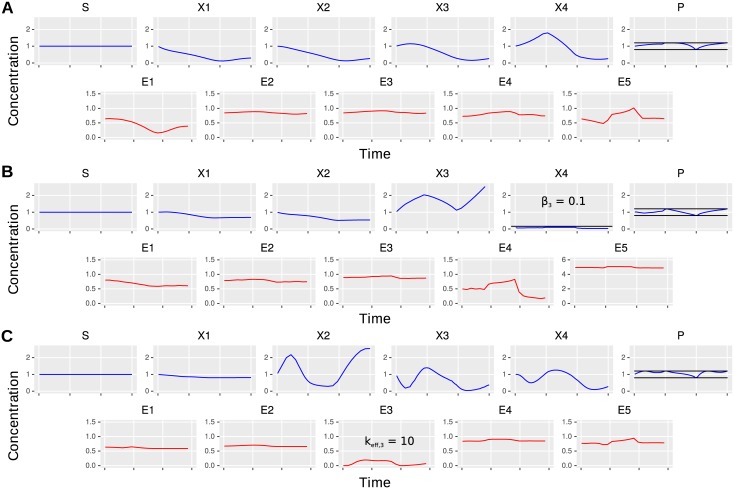
Comparison of optimal regulatory programs. (A) Optimal time-course of enzyme (in red) and intermediate (in blue) concentrations for *σ* = 1/30, uniform kinetics and no toxicity. (B) Optimal solution for the same parameters apart the low toxicity threshold *β*_4_ = 0.1 of intermediate *X*_4_. (C) Again the same parameters as in (A) apart the highly efficient third enzyme *k*_*eff,3*_ = 10. Threshold are depicted as black lines and the arbitrary time horizon ranging from 0 to 30 is discarded for lucidity.

For kinetic efficiency a clear tendency is visible that strongly regulated enzymes tend to be also highly efficient confirmed by a significant correlation (Spearman correlation *r* = 0.57, *P* < 1 ⋅ 10^−308^) of *k*_*eff*_ and fraction of regulatory effort. The correlation calculation includes all optimization results and shows that our observations are valid not only for the extreme cases (strong or weak), which are chosen to depict our results. Moreover, we observe a strong influence of intermediate toxicity on pathway regulation. The relation of toxicity thresholds, where low values correspond to high toxicity, and regulatory effort reveals that strongly regulated enzymes are followed by intermediates with low toxicity thresholds (Spearman correlation *r* = −0.21, *P* < 3 ⋅ 10^−13^ and Fig 3B and 3D). We see that the highly regulated enzyme can be further upstream of the toxic intermediate (see Fig 4C). In contrast to this, direct upstream intermediates have preferably high toxicity thresholds meaning a lower toxicity (Spearman correlation *r* = 0.49, *P* < 6 ⋅ 10^−74^ and Fig 3B and 3D). This observation can be explained by a regulatory program that shifts regulation to enzymes with less toxic upstream intermediates that can accumulate during regulation as we see in the time course of metabolite concentrations (compare Fig 4A and 4B).

Both observations are less pronounced in the scenario with high enzyme costs since the ensuing pervasive regulation leads to a coordinated regulation of all enzymes and thus a reduced accumulation of intermediates. This is most obvious by smaller median differences of the medians of parameter distributions between strongly and weakly regulated pathway models compared to the scenario of low enzyme costs (Fig 3B and 3D and S1 Fig).

To illustrate the influence of toxicity constraints on pathway regulation, we compared the optimal sparse regulation of a pathway without toxicity constraints and uniform enzyme efficiency (Fig 4A) to cases with a tight constraint on the concentration of the last intermediate (Fig 4B) and a highly efficient third enzyme (Fig 4C).

The dynamics for the reference parameter set involves regulation at the first and last position to optimally balance product drain. This leads to an accumulation of the last intermediate *X*_4_, which is used as a buffer for the product (Fig 4A). The dynamics change completely if this intermediate is toxic (Fig 4B). In this case, the optimal regulatory target is the fourth enzyme and intermediate *X*_3_ accumulates, since the accumulation of intermediate *X*_4_ is avoided due its toxicity. Analogously, if the third enzyme is much more efficient, we see a shift in the regulatory effort toward this enzyme independently of the toxicity of intermediates (Fig 4C).

In order to verify the generality of our results, we applied the same analysis to models of shorter and longer linear pathways, pathways with product inhibition, reversible reactions and changes in rates of dilution of the product of the pathway. We could confirm our observations also after these modifications to our basic model (see S2 Text).

### Intermediate toxicity shapes the regulation of prokaryotic metabolic pathways

We validated the predictions of the optimization in an analysis of regulation in the metabolic networks of more than 5000 prokaryotes listed in the BioCyc database. Apart from the collection of metabolic pathways, this database contains predictions on the operonic structure of the listed organisms which allows us to reliably infer promoter lengths and hence regulatory effort targeted at individual genes in each organism. In the metabolic networks, linear pathways were identified and combined with data of predicted regulatory effort (promoter length, PTM-sites), intermediate toxicity and kinetic parameters (for *Escherichia coli*). Further, to make pathways comparable and to match our model, we defined five pathway intervals to which enzymes at different pathway positions were assigned (see Methods).

**Fig 5:**
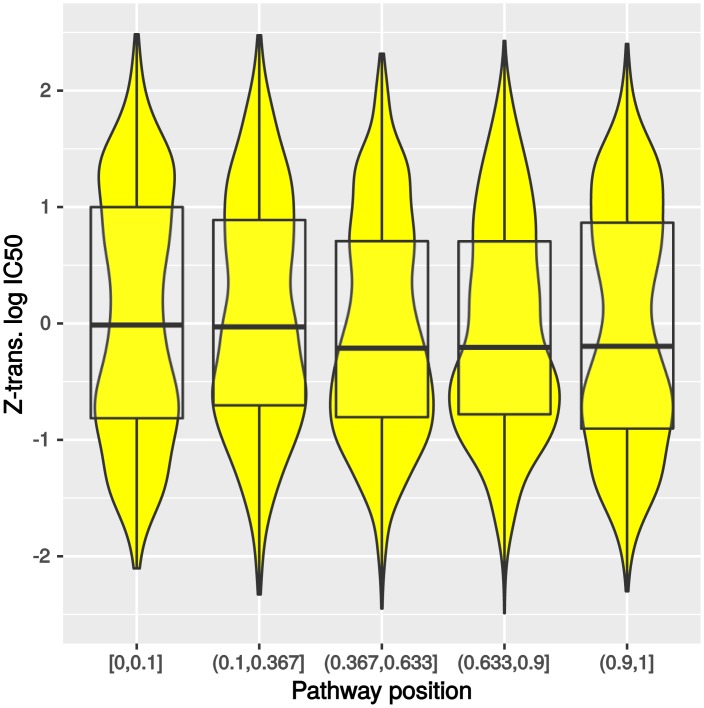
Distribution of intermediate toxicity for each pathway interval of all compounds listed in metacyc (lower values indicate higher toxicity).

In a first step, we investigated the distribution of intermediate toxicity across the pathway positions collected in the MetaCyc database (see Fig 5). We observed that substrates and products of pathways have slightly higher IC50 values than intermediates of pathways (first and last interval compared to intervals in between, Mann–Whitney–Wilcoxon test, *P* = 0.019), that is they are less toxic. From an evolutionary point of view, this observation makes intuitively sense since substrates and products of pathways are usually present in higher concentration than intermediates that are only transiently formed [29]. This suggests that the structure of metabolism has been shaped by avoiding toxic intermediates as substrates or products of pathways. On the other hand, substrates and products of pathways might also be less toxic because cells had to adapt to their higher concentration in contrast to intermediates that are present in smaller quantities. Other optimization approaches suggested that the limited solvent capacity leads to low concentrations of intermediates to avoid high osmotic pressure in the cell [30].

Similar to the analysis of the optimization results, we separated data sets into cases of strong and weak regulation at individual pathway positions. To this end, we defined for each pathway interval the 10% of enzymes with highest regulatory effort as strongly regulated and the 10% of enzymes with smallest regulatory effort as weakly regulated (see Methods). Since the optimality principle of avoiding toxic intermediates was more pronounced in the case of low enzyme costs in the simulations, we focused the validation on sparsely regulated metabolic pathways. The strongly regulated enzymes represent targets of regulation and the toxicity is compared of upstream intermediates, direct and further downstream intermediates with weakly regulated pathways at this position. The resulting data set compromises 1,012,285 reactions with enzymes as data points from 214,550 defined linear metabolic pathways across 5292 organisms (see S1 Table).

**Fig 6:**
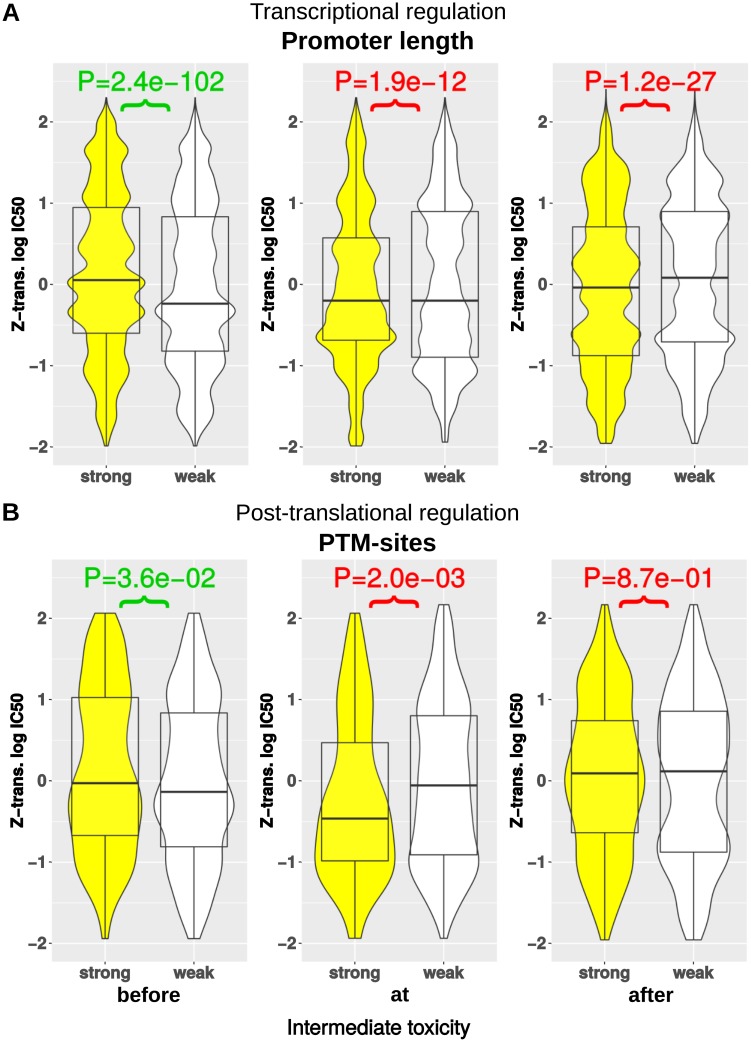
Comparisons of intermediate toxicity distributions before (left), at (middle) and after strongly regulated enzymes for (A) transcriptional regulation and (B) post-translational regulation. Yellow color indicates toxicity distributions for strongly regulated enzymes, and white color the corresponding toxicity distributions for direct downstream intermediates of weakly regulated enzymes (lower values indicate higher toxicity).

In accordance with the optimization results, we observed a significantly lower toxicity of intermediates before the strongly regulated enzymes (Mann–Whitney–Wilcoxon test, *P* < 1 ⋅ 10^−16^, see Fig 6). A closer inspection of the corresponding intermediates revealed that the direct upstream intermediates of the strongly regulated enzymes have a lower toxicity (see S2 Fig). This confirms that intermediates prior to strongly regulated enzymes are used as a buffer for varying demands of product concentrations and therefore have on average lower toxicity.

Further, our optimization results are moreover supported by the observation that the downstream intermediates of strongly regulated enzymes are significantly more toxic (Mann–Whitney–Wilcoxon test, *P* = 1.9 ⋅ 10^−12^, see Fig 6). The use of logarithmized IC50 and the subsequent Z-transformation leads to differences of toxicity in the magnitude of 10^−1^. In this case a difference of 10^−1^ corresponds to 10% of the variance of the toxicity across the pathway, where IC50 values can easily vary from 10^−4^ *g l*^−1^ for toxic metabolites to 10^2^ *g l*^−1^ for non-toxic metabolites. The positional analysis of intermediate toxicity moreover revealed that highly toxic intermediates are not always directly produced by the strongly regulated enzyme, but can also occur further downstream (see S2 Fig). As we have seen in our optimization results this can be explained by the influence of enzyme efficiency and the preference of highly efficient enzymes as main regulatory targets.

### Efficient enzymes are main targets of transcriptional regulation in *Escherichia coli*

In the optimization results, we observed a strong influence of kinetic efficiency of enzymes in combination with intermediate toxicity on the optimal regulatory strategy. Since a large-scale data set for all prokaryotes is not available for kinetic parameters and prediction, we verified our hypotheses in *Escherichia coli*.

We retrieved all experimental determined values of *k*_*cat*_ and *K*_*M*_ for *Escherichia coli* from the Brenda database [25] and obtained 266 enzymes with *k*_*cat*_ and *K*_*M*_ values as data points. We reduced the number of intervals dividing the pathway positions (first, middle, last) and mapped the properties.

**Fig 7:**
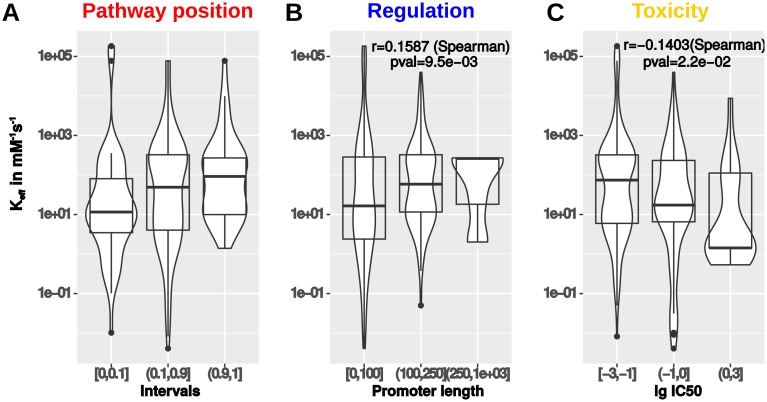
Relation of kinetic efficiency and pathway position (A), regulation (B) and product toxicity (C, lower values indicate higher toxicity) in *Escherichia coli*.

Interestingly, the distribution of *k*_*eff*_ shows that the initial enzymes of pathways (11.6 *mM*^−1^ *s*^−1^ average efficiency) tend to be much less efficient than the terminal enzymes (94.1 *mM*^−1^ *s*^−1^ average efficiency) in a linear pathway (Mann–Whitney–Wilcoxon test, *P* = 5.1 ⋅ 10^−3^, see Fig 7A). This can be explained by the need for a more precise regulation at the terminal step of pathways to fine-tune the synthesis of the product of the pathway according to cellular needs as we reported previously [10, 15]. The distribution of intermediate toxicity is inverse with a lower log IC50 for late intermediates and a higher log IC50 for early intermediates suggesting a relation between intermediate toxicity and kinetic efficiency. This is confirmed by the significant correlation between kinetic efficiency and toxicity (Spearman correlation *r* = −0.14, *P* = 0.022, see Fig 7C) and is linked to the correlation of kinetic efficiency and promoter length (Spearman correlation *r* = −0.16, *P* = 0.009, see Fig 7B). Both relationships between intermediate toxicity, kinetic efficiency and regulation are in line with our optimization results and show that there is a evolutionary selection for efficient enzymes as strongly regulated enzymes optimally controlling toxic intermediate accumulation. Further, the results show a link to the hypothesis of retro-evolution of pathways [31], which assumes that pathways are extended by the invention of enzymes which are able to synthesize the product from more distant intermediates and therefore final and evolutionary older enzymes have a higher efficiency. Additionally, the same is true for the toxicity of intermediates, because we see that late intermediates are more toxic than the early intermediates (see Fig 5), which can be seen as evolutionary newer sources for the required product to circumvent toxic intermediates.

### Using intermediates as endogenous antimicrobials: Acetate as a by-product of lipid *IV*_*A*_ biosynthesis in *Escherichia coli*

Our results about the interplay of intermediate toxicity, kinetic efficiency and regulation of linear metabolic pathways in prokaryotes have demonstrated the importance of fine tuned regulatory programs for controlling metabolic pathways to avoid the production of toxic intermediates. In the other direction, this suggests that we could use information about strongly regulated enzymes to infer metabolites whose production and consumption is under tight control and hence they might potentially be harmful to the cell. Examples for antimicrobials that follow this principle are antibiotics targeting late steps in the teichoic acid biosynthesis in gram-positive bacteria [32]. In this case, late intermediates are accumulated increasing the osmotic stress and enhancing the antimicrobial effect [33].

**Fig 8:**
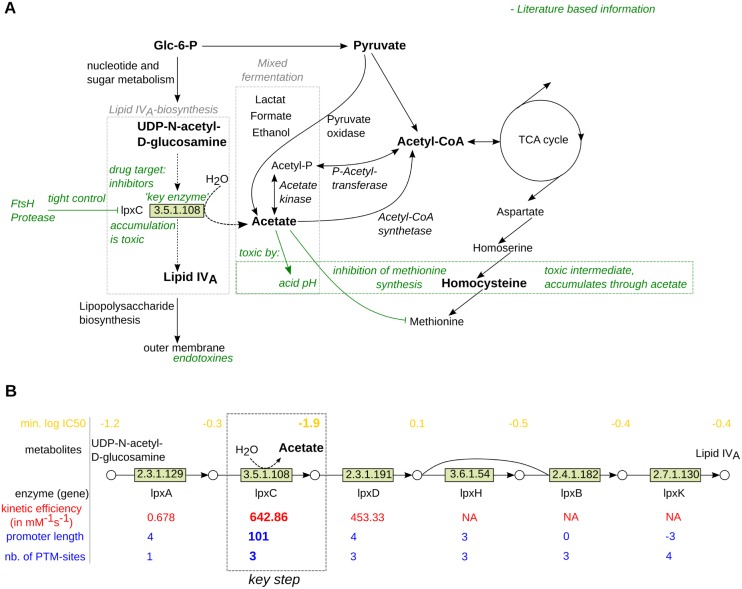
(A) Excerpt of metabolic context of acetate and lipid *IV*_*A*_ biosynthesis. The green box highlights mechanisms of acetate toxicity. (B) lipid *IV*_*A*_ biosynthetic pathway including key characteristics (lower log IC50 values indicate higher toxicity).

An interesting example is provided by lipid *IV*_*A*_ biosynthesis which produces not only lipopolysaccharides precursors as components of the cell wall of *E. coli* but also the toxic byproduct acetate (see Fig 8). Despite the ability of *Escherichia coli* to utilize acetate as a carbon source, higher concentrations of acetate (> 8 *mM*) are reported to inhibit growth by various mechanisms (see green box in Fig 8A) [34].

In agreement with our predictions, the acetate producing enzyme LpxC (UDP-3-O-acyl-N-acetylglucosamine deacetylase) has a very high catalytic efficiency (642.86 *mM*^−1^ *s*^−1^) and the highest promoter length (101 bp) within the pathway (see Fig 8B). This data confirms the description in the literature that this step is the key step of lipid *IV*_*A*_ biosynthesis and is tightly controlled by the ATP-dependent metalloprotease FtsH [35]. The gene *lpxC* is located in an operon together with several genes controlling cell division. However, *ftsH* is transcribed separately providing a precise regulation and prevents accumulation or depletion of the key enzyme which has been described as lethal in previous studies [35, 36]. However, previous research focused on the discovery of inhibitors to block lipid *IV*_*A*_ biosynthesis and subsequently weakening the membrane of prokaryotes. Thus, our results suggest that either inhibiting acetate consuming enzymes or overproducing LpxC represent routes to use acetate as an endogenous antimicrobial. However, overproducing LpxC might have side-effects due to its role in cell wall biosynthesis.

Key mechanisms of acetate toxicity in *E. coli* are summarized in Fig 8A. Acetate is a side-product during mixed acid fermentation where glucose is converted to lactate, acetate, succinate and formate as well as ethanol in anaerobic conditions [37]. Moreover, acetate is a major byproduct during growth on excess glucose, referred to as overflow metabolism [38], and is produced not only via degradation of acetyl-CoA but also directly by pyruvate oxidation at low growth rates [39]. The mechanism behind acetate toxicity is based on several factors. First of all, acetate lowers the pH in the cell [34], which impacts many cellular processes like protein folding or ion transport [40]. Additionally, acetate inhibits the production of methionine via homocysteine which then accumulates and is also reported as a toxic intermediate [34]. To reduce the concentration of acetate the cell can export acetate [41], which leads to acetate accumulation in the environment, or convert it to acetyl-CoA, which can be oxidized in the tricarboxylic acid cycle [42]. Acetate is converted into acetyl-CoA either via acetate kinase and phosphotransacetylase at high concentrations of acetate, or through the acetyl-CoA synthetase during late exponential phase [43]. Therefore both pathways and its enzymes are potential targets for inhibition to induce an accumulation of acetate.

## Discussion

In the present study, we used dynamic optimization to elucidate the influence of toxic intermediates on the regulation of linear metabolic pathways. Previous studies showed that toxic intermediates have a high impact on the yield of pathways [44] and accumulation is prevented by feedback mechanisms [34, 45] as well as operonic organization [46].

Our optimization results disclose that sparsely regulated pathways with strongly regulated enzymes at the first and last position use late intermediates to buffer the varying demand of product concentrations. However, if these intermediates are highly toxic they cannot be used as buffer and regulation needs to target enzymes further upstream. The optimization showed that especially kinetic efficient enzymes are the targets of regulation and hence, the position of the strongly regulated enzyme is strongly influenced by the interplay of intermediate toxicity and kinetic characteristics of a pathway. Together with other factors, like pathway topology or enzyme costs [10, 15], dynamic optimization allowed us to disentangle the influence of many features on pathway control thereby expanding the classical view of pathway regulation like key enzymes and rate limiting steps.

We confirmed the predicted optimality principles by an analysis of pathway regulation on the transcriptional and post-translational level independently across all prokaryotes listed in the BioCyc database by relating enzyme regulation with intermediate toxicity estimations. Further, we showed for *E. coli* that kinetic properties of enzymes correlate with regulatory effort and intermediate toxicity. Our observation have been proven to be robust to changes of our optimization model and validation process by varying pathway characteristics like length, inhibition kinetics and product dilution dynamics.

The findings provide further insight in the complex strategies behind pathway regulation. In recent years, it became more and more apparent that regulation of pathways cannot be described only by metabolic control analysis, feedback mechanisms and other non-dynamical approaches. Our disclosure of toxic intermediates as additional characteristic determining the positions of regulation extends the understanding of pathway regulation and reveals new opportunities for optimizing pathway yield or find targets for antimicrobial interventions.

As shown for the case of lipid *IV*_*A*_ of *E. coli*, toxic intermediates and their producing enzymes are targets for antimicrobial interventions. The deliberate deregulation of a pathway can possibly lead to an accumulation of toxic intermediates like acetate, which act as a endogenous antibiotic and kills the cell. Through an analysis of regulatory effort, intermediate toxicity as well as catalytic efficiency, targets can be identified on a large scale supporting the discovery of new antibiotic drugs. This approach could provide more and organism specific targets than the focus of most antibiotics to interfere with the cell wall and its synthesis or the synthesis of DNA and protein [47].

Despite the more complex regulation in eukaryotes and different cellular preconditions like the decoupling of transcription and translation, we think that the same optimality principles are valid. This has been shown, for instance, in an metabolic engineering approach in yeast where the avoidance of the accumulation of toxic intermediates allowed for an increased production of a desired product through improved cell viability [44].

Especially, our approach and these investigations can be applied to eukaryotic pathogens like the fungal species *Candida albicans* or *Aspergillus fumigatus*. For instance, the ergosterol pathway in fungi is attacked by Amphotericin B, specifically ERG3, synthesized by a gene encoding sterol Delta(5,6)-desaturase, so creating an important intermediate of ergosterol pathway [48]. Also azols hit this target leading to the disruption of the pathway and intermediate accumulation, subsequently stopping growth of *Candida albicans* [48]. However, the opposite strategy, as explored here, to push ERG3 and other enzyme action such that more toxic intermediates are accumulating has not been investigated for drug development. For instance, ERG11 of *Candida albicans is* a potential target leading to accumulation of intermediates, which was also observed under anaerobic conditions where ERG3 is non-functional [48]. As all these enzymes are not present in humans, we consider this as an interesting alternative to explore, not only for *Candida* but also for *Aspergillus fumigatus*. Both fungal pathogens are the main causes of life-threatening invasive mycoses [49] and drug development is difficult due to the closer relationship between human and fungi compared to prokaryotes.

## Supporting information

S1 Text ODE system. Detailed description and mathematical formulation of ODE system and optimization problem.
(PDF)

S2 Text Robustness of optimality principles. Analysis of pathway models considering product inhibition, reversible reactions, different pathway lengths and random dilution rates.
(PDF)

S1 Fig Positional relation of regulation, kinetic efficiency and intermediate toxicity from optimization. Influence of parameters (yellow: toxicity, red: kinetic efficiency) on the regulatory strategy and the position of strongly regulated enzymes (blue) for low enzyme costs (A) and high enzyme costs (B). For each strongly regulated enzyme position (row) arrows indicate the difference of medians of toxicity (*β*) and kinetic efficiency (*k*_*eff*_) between strong and weak regulation at each position. Arrow sizes are scaled to the maximal median difference depicted on the right column.
(PDF)

S2 Fig Positional relation of regulation and intermediate toxicity in prokaryotes. Relation of regulation (A) promoter length and (B) PTM-sites with toxicity of intermediates. Arrows pointing upwards showing higher toxicity thresholds (lower toxicity) and arrows pointing downwards vice versa.
(PDF)

S1 Table Regulation and toxicity of linear pathways across prokaryotic species. Tables of promoter length and PTM-sites, as well as IC50 of downstream intermediates for each defined reaction of linear metabolic pathway.
(7Z) Click here for additional data file.
