# Supplementary material for: Optimality principles reveal a complex interplay of intermediate toxicity and kinetic efficiency in the regulation of prokaryotic metabolism
Source: PLoS Comput Biol. 2017 Feb 17;13(2):e1005371. doi: 10.1371/journal.pcbi.1005371 (PMC5315294; doi:10.1371/journal.pcbi.1005371)
Supplement: S1 Text — Detailed description and mathematical formulation of ODE system and optimization problem. (PDF) [file pcbi.1005371.s001.pdf]

S1 ODE system

The concentrations of all metabolites are combined in the vector $m(t)$. The derivative of metabolite concentrations $\dot{m}(t)$ is calculated by the velocity of production deducting the velocity of consumption:

$$m(t) = \begin{pmatrix} s(t) \\ x_1(t) \\ x_2(t) \\ x_3(t) \\ x_4(t) \\ p(t) \end{pmatrix} \qquad \dot{m}(t) = \begin{pmatrix} 0 \\ v_1(t) - v_2(t) \\ v_2(t) - v_3(t) \\ v_3(t) - v_4(t) \\ v_4(t) - v_5(t) \\ v_5(t) - v_g(t) \end{pmatrix} \tag{1}$$

The substrate is considered to be buffered and therefore has a constant concentration of $s(t) = 1$ (arbitrary units). The initial concentration of the product is $p(0) = 1$ and the initial concentration of the metabolites $x_i(0)$ depends on their toxicity (see next paragraph).

The kinetics of the reactions are modelled by irreversible Michaelis-Menten kinetics with the parameters $k_{cat,j}$ and $K_{m,j}$ and controlled by enzyme concentrations $e_j(t)$ as the control variables:

$$\begin{aligned} v_1(t) &= \mathbf{e_1(t)} \frac{s(t) \cdot k_{cat,1}}{K_{m,1} + s(t)} \\ v_j(t) &= \mathbf{e_j(t)} \frac{x_{j-1}(t) \cdot k_{cat,j}}{K_{m,j} + x_{j-1}(t)}, \; j = 2, ..., 5 \end{aligned} \tag{2}$$

The dilution rate, v_g, is a self-defined function with changing rates over time, described by three rate factors $g_{1...3}$:

$$v_g(t) = \begin{cases} g_1 = 0.3, & t < 10 \\ g_2 = 0.5, & 10 \leq t < 20 \\ g_3 = 0.1, & 20 \leq t \leq 30 \end{cases} \tag{3}$$

or randomized with $g_i \in [0, 1]$ for robustness analysis.

The toxicity has also an influence on the initial concentrations of metabolites, since their concentrations might exceed the toxicity threshold when arbitrarily set to one. Similar to the original implementation, we assume intermediate concentrations to sum to 4 (arbitrary units). For toxicity thresholds β_i smaller than an initial concentration of 1, initial metabolite concentrations are distributed equally over less toxic metabolites.
