# Supplementary material for: Optimality principles reveal a complex interplay of intermediate toxicity and kinetic efficiency in the regulation of prokaryotic metabolism
Source: PLoS Comput Biol. 2017 Feb 17;13(2):e1005371. doi: 10.1371/journal.pcbi.1005371 (PMC5315294; doi:10.1371/journal.pcbi.1005371)
Supplement: S2 Text — Analysis of pathway models considering product inhibition, reversible reactions, different pathway lengths and random dilution rates. (PDF) [file pcbi.1005371.s002.pdf]

S2 Robustness of optimality principles.

Since our model of a metabolic pathway is a simplification, we explored the robustness of our discovered optimality principles by varying the model to describe product inhibition, reversibility of reactions, random dilution rates or different pathway lengths. Each feature is independently analyzed for its influence on the results.

Variation of pathway length

The average lengths of the considered linear metabolic pathways in MetaCyc is 5.079, but can vary from the smallest linear pathway of three (shorter pathways are note considered as linear pathways in this study) to a maximal length of 92 for the mycolate biosynthesis. To elucidate the influence of pathway lengths, the same kinetics as described in S1 were used, but with three, respectively, seven enzymes. Following the same analysis of results as in the main text, we depict the optimal regulation of a shorter (Fig. 1) and a longer pathway (Fig. 2).

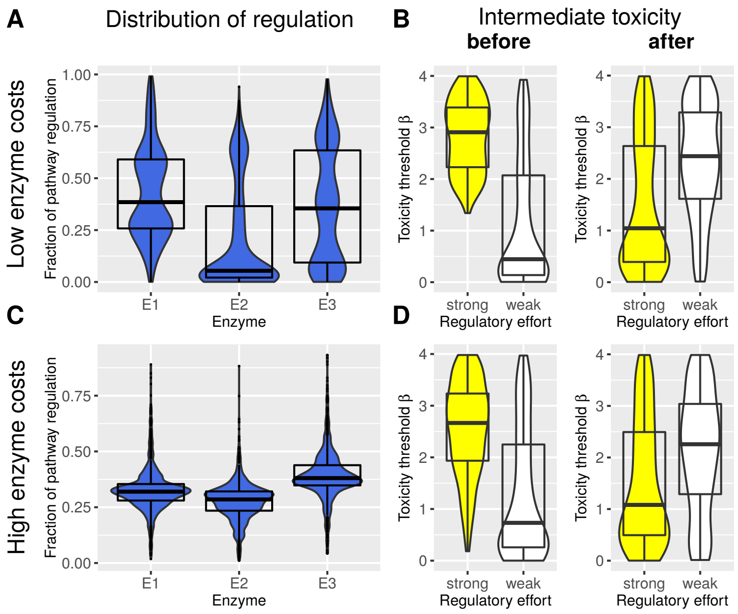

Figure 1: Pathway length: 3. Relation of regulatory effort and intermediate toxicity for low (A,B) and high enzyme costs (C,D). (A,C) The fraction of regulation is displayed in blue for each enzyme. (B,D) The intermediate toxicity is depicted in yellow (white) for the case of a highly (lowly) regulated enzyme and the toxicity threshold of intermediates before and after.

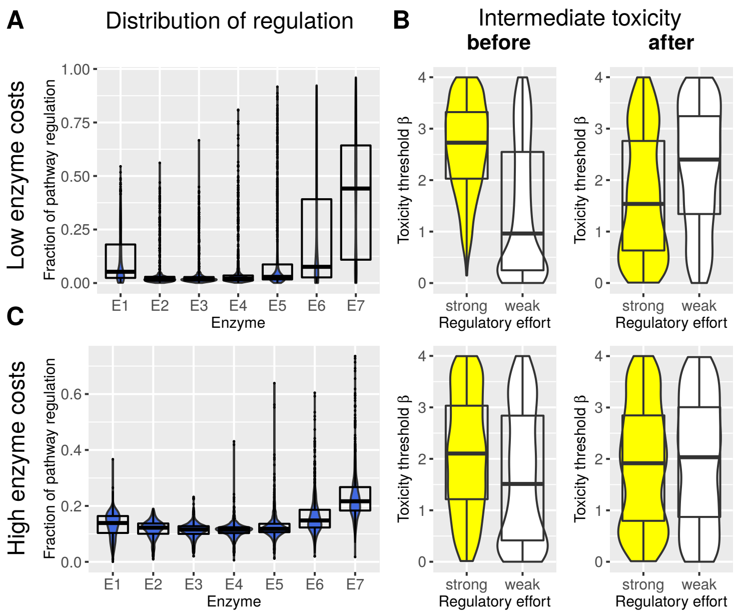

Figure 2: Pathway length: 7. Relation of regulatory effort and intermediate toxicity for low (A,B) and high enzyme costs (C,D). (A,C) The fraction of regulation is displayed in blue for each enzyme. (B,D) The intermediate toxicity is depicted in yellow (white) for the case of a highly (lowly) regulated enzyme and the toxicity threshold of intermediates before and after.a

For both cases the preferred regulatory strategy involves mainly the first and the last enzymes. Also intermediate toxicity is lower before the highly regulated enzymes and higher after the flux determining enzyme. As seen for the pathway length of five the observations are less pronounced for higher enzyme costs.

Because our validation process bins the different linear metabolic pathways of prokaryotes into five intervals, we repeated the analysis for a case of three and a case of seven bins. As expected the binning into three intervals (see Fig. 3) is not consistent with our results for more intervals. Since the pathways are mostly longer, the first and third enzyme are the targets of sparse regulation and a single intermediate enzyme is not suffcient to resolve the relation of toxic intermediates and regulation. In contrast to this, the binning into seven intervals is able to show similar results as we see for a number of five intervals (see Fig. 4).

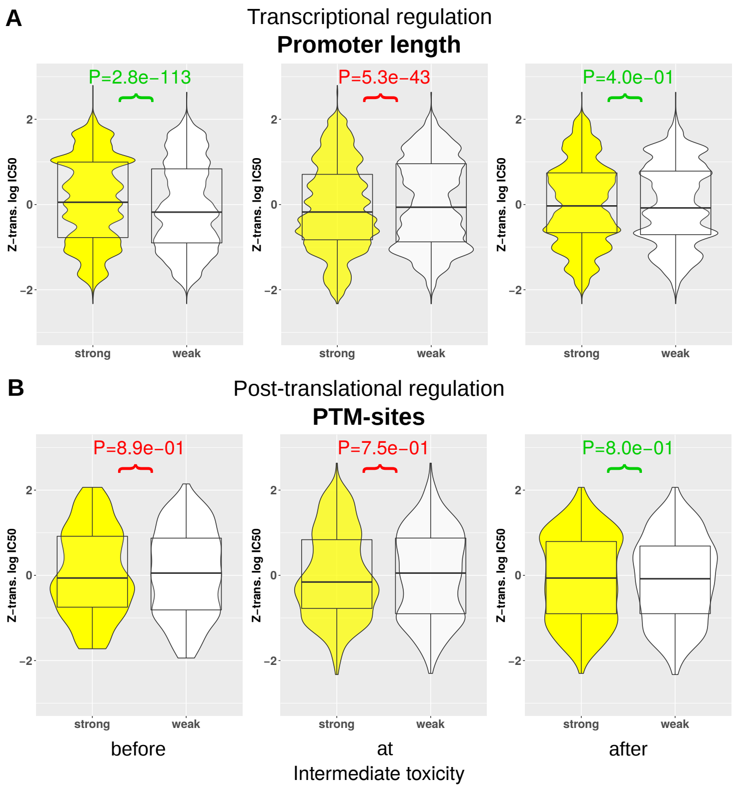

Figure 3: Pathway length: 3. Comparisons of substrate toxicity distributions before (left), at (middle) and after strongly regulated enzymes for (A) transcriptional regulation and (B) post-translational regulation.

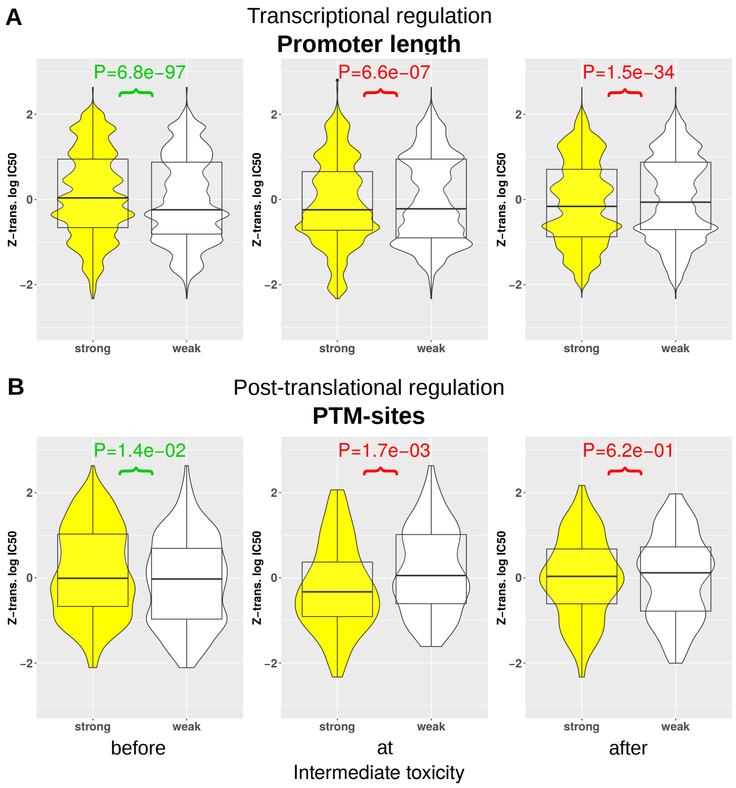

Figure 4: Pathway length: 7. Comparisons of substrate toxicity distributions before (left), at (middle) and after strongly regulated enzymes for (A) transcriptional regulation and (B) post-translational regulation.

Introduction of product inhibition

Product inhibition is a common feature of metabolic pathways and could potentially alter the optimal regulatory strategy. To implement the repression of a pathway flux by product inhibition we introduced competitive inhibition in v_1 as previously [1] with the repression constant $k_{r,1} \in [0, 2]$:

$$v_1(t) = \mathbf{e_1(t)} \frac{s(t) \cdot k_{cat,1}}{K_{m,1} \cdot (1 + \frac{p(t)}{k_{r,1}}) + s(t)} \tag{1}$$

The optimization reveals again similar regulatory strategies involving the first and last enzymes, as well as the relation of intermediate toxicity and regulatory effort (see Fig. 5).

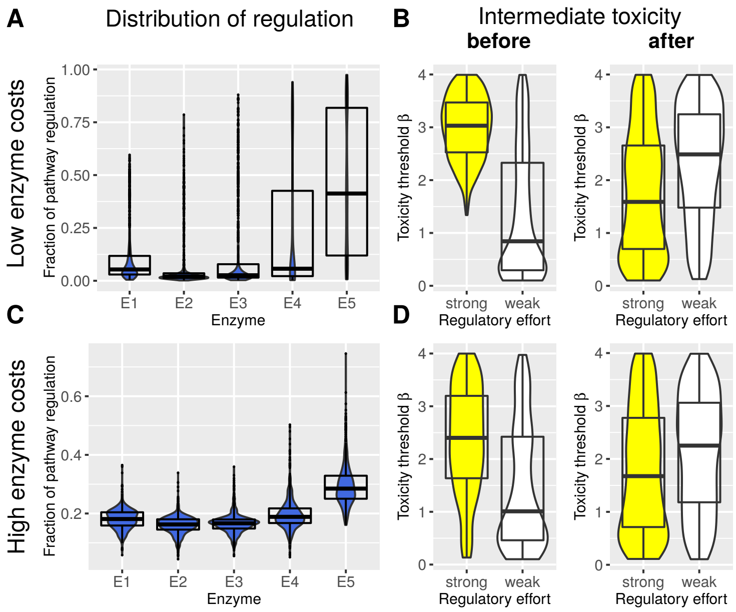

Figure 5: Product inhibition. Relation of regulatory effort and intermediate toxicity for low (A,B) and high enzyme costs (C,D). (A,C) The fraction of regulation is displayed in blue for each enzyme. (B,D) The intermediate toxicity is depicted in yellow (white) for the case of a highly (lowly) regulated enzyme and the toxicity threshold of intermediates before and after.

Randomization of dilution rate

The dilution rate is modeled to have a mid-, high- and low-level demand to simulate different types of product demand. To test the influence of dilution rate on our results, we randomized growth rates g_1,g_2,$g_3 \in [0, 1]$:

$$v_g(t) = \begin{cases} g_1, & t < 10 \\ g_2, & 10 \leq t < 20 \\ g_3, & 20 \leq t \leq 30 \end{cases} \tag{2}$$

The results of optimization are similar to the non-randomized dilution rates (Fig. 6).

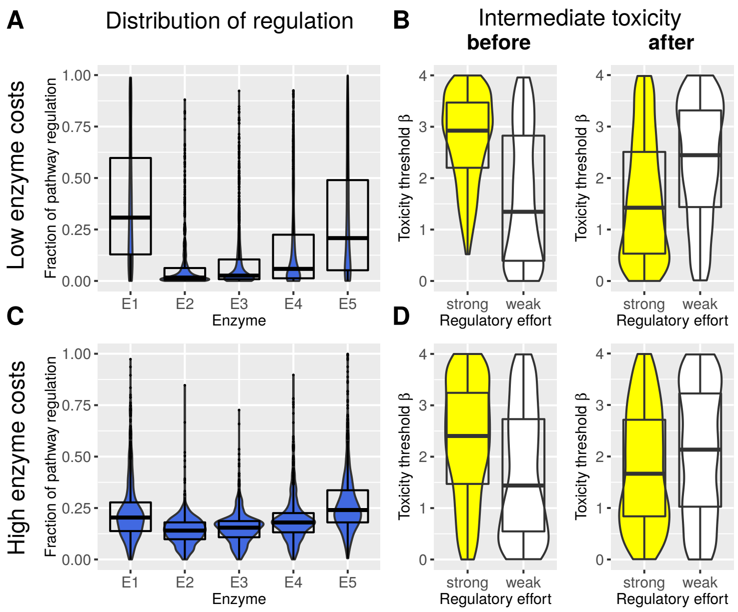

Figure 6: Randomized dilution rates. Relation of regulatory effort and intermediate toxicity for low (A,B) and high enzyme costs (C,D). (A,C) The fraction of regulation is displayed in blue for each enzyme. (B,D) The intermediate toxicity is depicted in yellow (white) for the case of a highly (lowly) regulated enzyme and the toxicity threshold of intermediates before and after.

Reversibility of enzymatic reactions

For simplicity the pathway model consists of irreversible reactions following Michaelis-Menten kinetics. To disclose possible differences of our results if reactions are reversible, we changed the rate laws to a reversible Michaelis-Menten kinetics with:

$$v_j(t) = \frac{e_j(t) \cdot (k^f_{cat,j} \cdot \frac{Y_j}{K^s_{m,j}} - k^r_{cat,j} \cdot \frac{Y_{j+1}}{K^p_{m,j}})}{1 + \frac{Y_j}{K^s_{m,j}} + \frac{Y_{j+1}}{K_{m,j}}} \tag{3}$$

$$j = 1, ..., 5$$

$$Y \in [s, x_1, x_2, x_3, x_4, p]$$

For analysis we used uniform kinetic parameters except the turnover rate of the reverse reaction $k^r_{cat,j}$ and assumed the intermediate x_4 to be slightly toxic $\beta_4 = 0.5$. For slow reversible reactions $k^r_{cat,j} < 0.5$ (see Fig. 7) we observe optimal regulatory programs mainly involving the enzyme before the toxic intermediate as we see for irreversible Michaelis-Menten kinetic. Because of the fast reverse reaction higher values of $k^r_{cat,j}$ lead first to optimal programs involving more enzymes upstream of the toxic intermediate (see Fig. 7D) and then to an infeasible optimization problem since either the product or the intermediate cannot be hold within the concentration bounds. Additionally, the required abundance of enzymes increases dramatically for reversible reaction since the control of enzymes vanishes with faster reverse reactions and the product can only be balanced by a fast conversion of the buffered substrate ($s(t) = 1$) to the diluted product. We conclude that in general the relation of regulation and toxicity of intermediates is similar for reversible Michaelis-Menten kinetics.

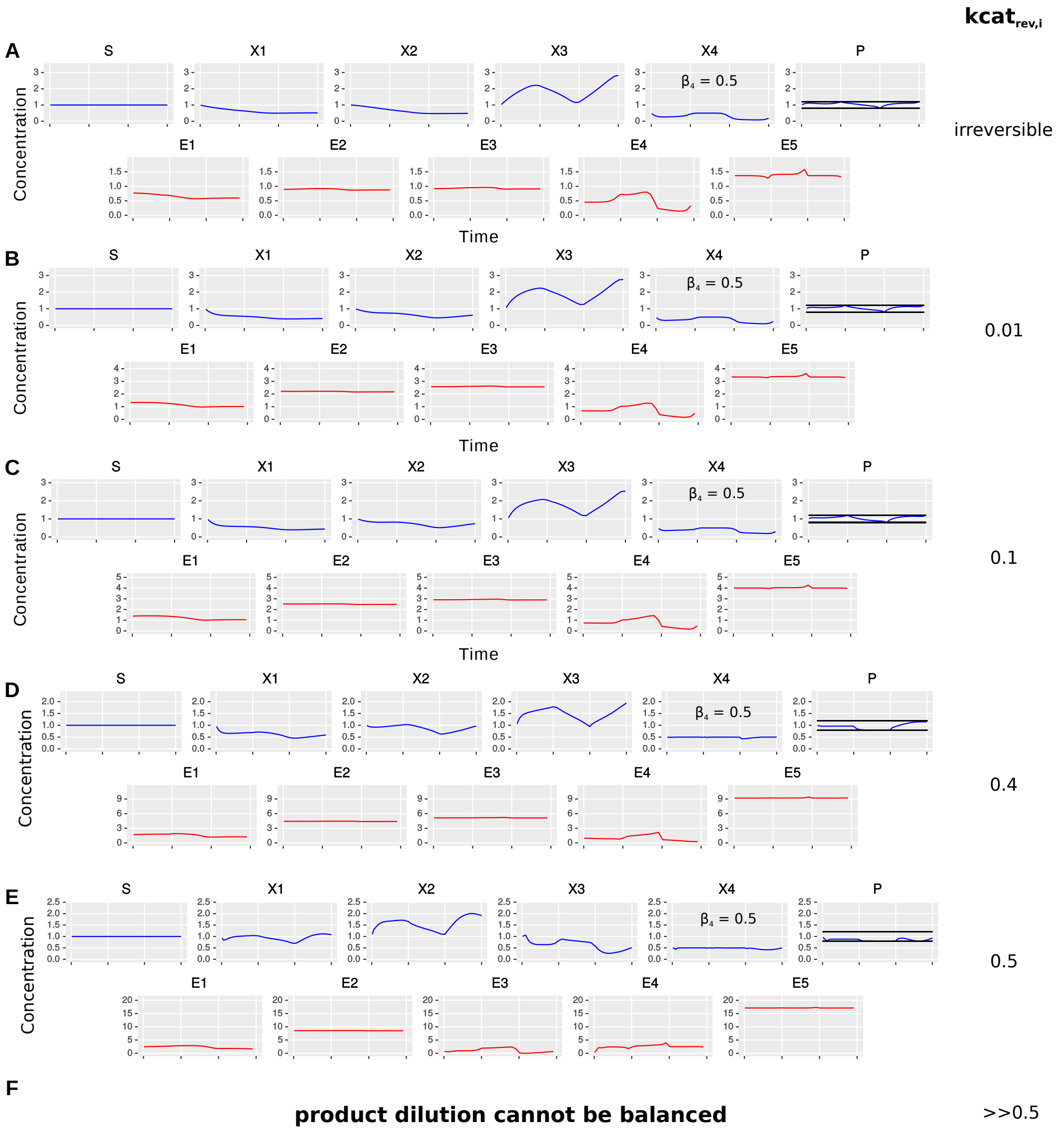

Figure 7: Comparison of optimal regulatory programs. (A)-(E) Solutions of optimization for irreversible reaction (A) as well as faster reverse reactions ($k^r_{cat,j}$). (F) For higher values $k^r_{cat,j} >> 0.5$ the optimization problem becomes infeasible.

References

[1] de Hijas-Liste GM, Balsa-Canto E, Ewald J, Bartl M, Li P, Banga JR, et al. Optimal programs of pathway control: dissecting the influence of pathway topology and feedback inhibition on pathway regulation. BMC Bioinformatics. 2015;16(1):163.
