# Supplementary material for: Optimality principles reveal a complex interplay of intermediate toxicity and kinetic efficiency in the regulation of prokaryotic metabolism
Source: PLoS Comput Biol. 2017 Feb 17;13(2):e1005371. doi: 10.1371/journal.pcbi.1005371 (PMC5315294; doi:10.1371/journal.pcbi.1005371)
Supplement: S1 Fig — Influence of parameters (yellow: toxicity, red: kinetic efficiency) on the regulatory strategy and the position of strongly regulated enzymes (blue) for low enzyme costs (A) and high enzyme costs (B). For each strongly regulated enzyme position (row) arrows indicate the difference of medians of toxicity (β) and kinetic efficiency (keff) between strong and weak regulation at each position. Arrow sizes are scaled to the maximal median difference depicted on the right column. (PDF) [file pcbi.1005371.s003.pdf]

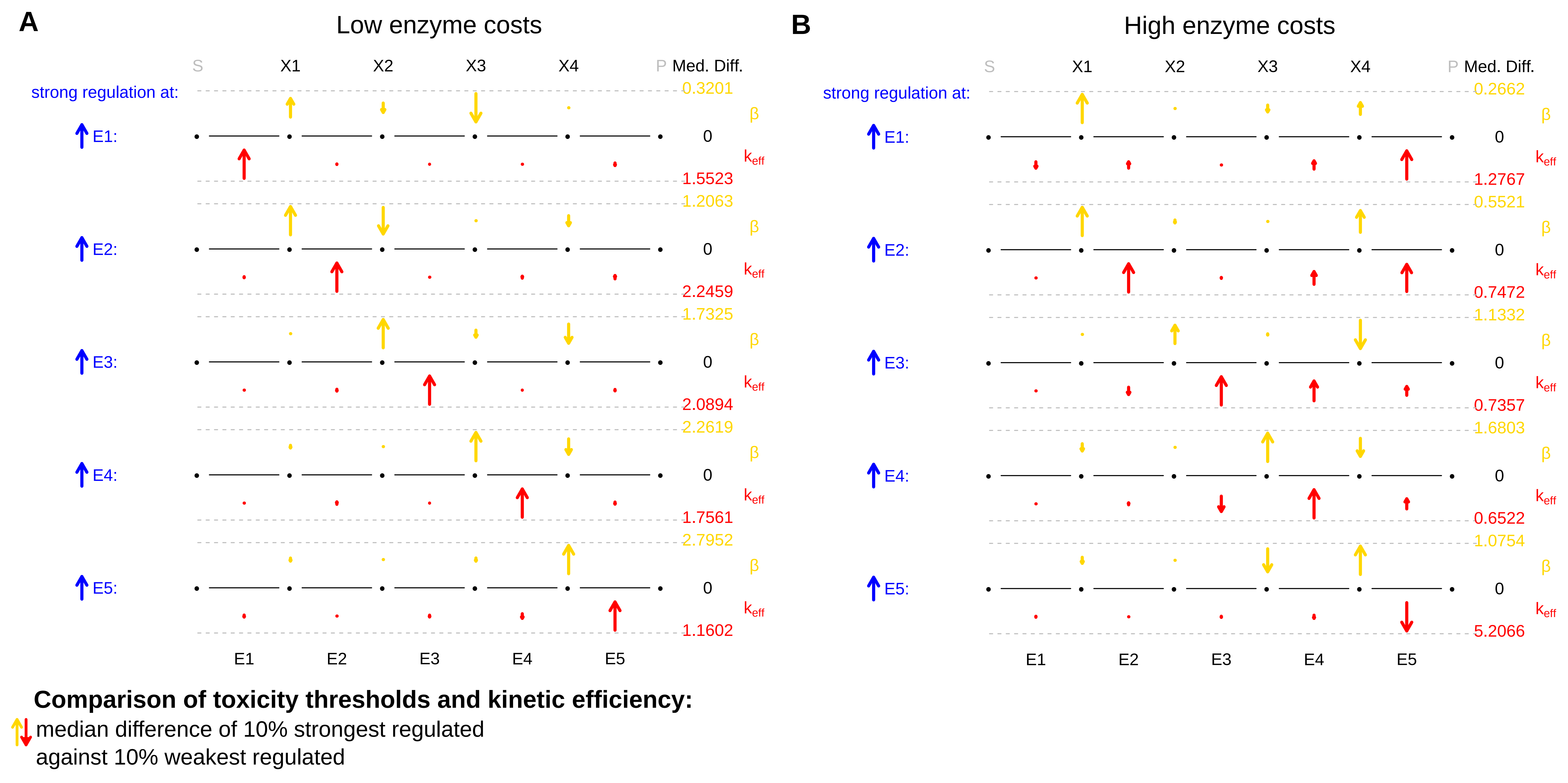

A
Low enzyme costs
B
High enzyme costs
S
X1
X2
X3
X4
P
Med. Diff.
strong regulation at:
E1:
E2:
E3:
E4:
E5:
β
k_{eff}
0
0.3201
1.5523
1.2063
2.2459
1.7325
2.0894
2.2619
1.7561
2.7952
1.1602
0.2662
1.2767
0.5521
0.7472
1.1332
0.7357
1.6803
0.6522
1.0754
5.2066
E1
E2
E3
E4
E5
Comparison of toxicity thresholds and kinetic efficiency:
median difference of 10% strongest regulated
against 10% weakest regulated
