# Supplementary material for: Optimality principles reveal a complex interplay of intermediate toxicity and kinetic efficiency in the regulation of prokaryotic metabolism
Source: PLoS Comput Biol. 2017 Feb 17;13(2):e1005371. doi: 10.1371/journal.pcbi.1005371 (PMC5315294; doi:10.1371/journal.pcbi.1005371)
Supplement: S2 Fig — Relation of regulation (A) promoter length and (B) PTM-sites with toxicity of intermediates. Arrows pointing upwards showing higher toxicity thresholds (lower toxicity) and arrows pointing downwards vice versa. (PDF) [file pcbi.1005371.s004.pdf]

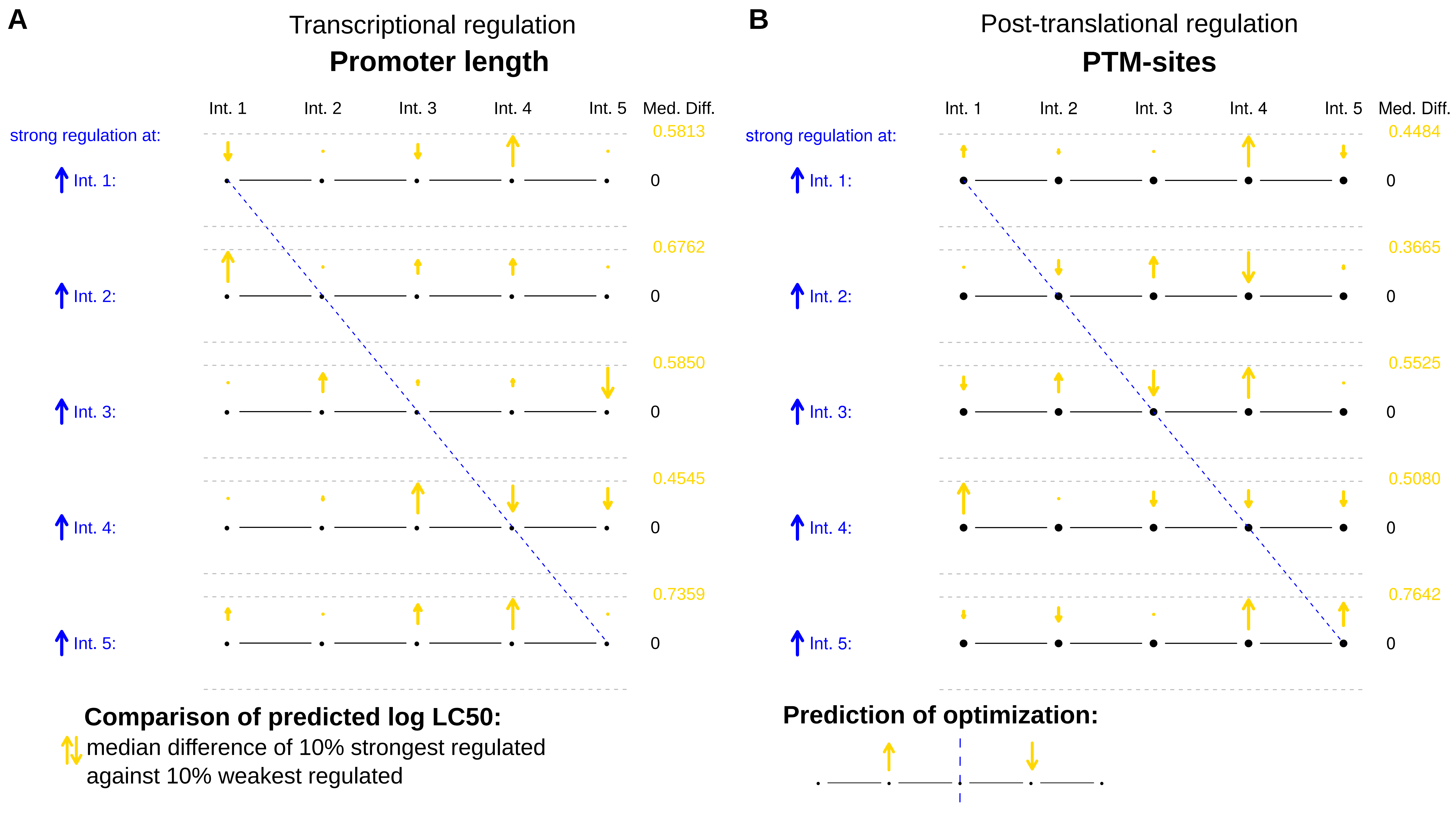

A
Transcriptional regulation
Promoter length
Int. 1
Int. 2
Int. 3
Int. 4
Int. 5
Med. Diff.
strong regulation at:
Int. 1:
Int. 2:
Int. 3:
Int. 4:
Int. 5:
0.5813
0.6762
0.5850
0.4545
0.7359
0
Comparison of predicted log LC50:
median difference of 10% strongest regulated against 10% weakest regulated
B
Post-translational regulation
PTM-sites
0.4484
0.3665
0.5525
0.5080
0.7642
Prediction of optimization:
